# Inflammatory bowel disease therapeutics: a bibliometric analysis of tofacitinib research in ulcerative colitis

**DOI:** 10.3389/fphar.2025.1570238

**Published:** 2025-04-01

**Authors:** Jianping Zhou, Yuting Xi, Yaping Zhang, Rui Zhang, Hao Fu, Ce Zhou

**Affiliations:** ^1^ Hospital of Chengdu University of Traditional Chinese Medicine, Chengdu, China; ^2^ Zigong Hospital of Traditional Chinese Medicine, Zigong, China

**Keywords:** tofacitinib, ulcerative colitis, bibliometric analysis, clinical response, remission, adverse event

## Abstract

**Background:**

Ulcerative colitis (UC) is a chronic inflammatory bowel disease affecting millions worldwide. Tofacitinib, an inhibitor of Janus kinase (JAK), has shown considerable potential as an effective treatment option for individuals suffering from moderate to severe UC, aiming to mitigate the risk of colectomy, hospitalization, and disease exacerbation.

**Methods:**

We conducted a comprehensive literature review from 2012 to 2024 to assess the study landscape of Tofacitinib in UC. Employing the Web of Science Core Collection database (WOSCC) and the bibliometric tool CiteSpace, we performed an bibliometric analysis to delineate disciplinary evolution and identify research hotspots within the UC Tofacitinib domain.

**Results:**

Our analysis extracted 406 UC Tofacitinib-related articles from WOSCC, indicating a growing body of literature. The United States and Europe are at the forefront of research maturity, with a significant contribution to the field. Here we show that multidisciplinary research is burgeoning, which is crucial for the advancement of UC Tofacitinib studies. We identified 13 highly cited documents and 10 co-cited documents, highlighting Tofacitinib’s prominence as a small molecule agent. Keyword analysis revealed that the intestinal barrier, clinical response, remission rate, and safety are the central themes of current research.

**Conclusion:**

By applying bibliometrics, citation analysis, and knowledge mapping, this study provides a snapshot of the current state and trajectory of Tofacitinib research in UC. We have elucidated the knowledge lineage in this field, offering insights that can inform both ongoing and future research endeavors. Our findings underscore the importance of multidisciplinary collaboration in advancing UC therapeutic strategies.

## 1 Introduction

Ulcerative colitis (UC) is a chronic inflammatory bowel disease (IBD) affecting the colon and rectum (Kobayashi et al., 2020a). While it can occur at any age, it is most prevalent among individuals aged 20 to 40. Approximately 1.5 million people in North America have been diagnosed with UC (Voelker, 2024). By 2023, its global prevalence was estimated at 5 million cases (Le Berre et al., 2023). UC is mainly characterized by abdominal pain, chronic diarrhea, rectal bleeding, and weight loss (Wangchuk et al., 2024). Inflammation is limited to the mucosal layer of the bowel and is marked by reduced microbiota diversity and thinning of the mucosal layer, resulting in barrier dysfunction (Kobayashi et al., 2020b). Although mucosal healing is associated with sustained remission, relapses with persistent disease activity have been observed in both clinical practice and pivotal trials (Krugliak et al., 2022). Hospitalization rates for IBD are stabilizing in stage III countries but are rising rapidly in stage II newly industrialized countries, increasing the burden on global healthcare systems (Buie et al., 2023). Current management focuses on inducing and maintaining remission, defined as symptom resolution and endoscopic healing (Ungaro et al., 2017). Treatment options include 5-aminosalicylic acid medications, steroids, and immunosuppressants. However, their effectiveness is constrained, with clinical trials showing response rates between 30% and 60%. Within 5 years of diagnosis, around 20% of patients with UC are hospitalized, and 7% undergo colectomy (Gros and Kaplan, 2023).

Tofacitinib is an oral small-molecule Janus kinase (JAK) inhibitor extensively studied for treating autoimmune diseases such as rheumatoid arthritis, systemic sclerosis, herpetic pemphigoid, and polyarteritis nodosa (Fan and Wang, 2023; Khanna et al., 2022; Rimar et al., 2022; Smolen et al., 2023). The JAK family includes four tyrosine kinases: JAK1, JAK2, JAK3, and non-receptor tyrosine kinases, which activate signal transducers and activators of transcription (STATs) via autophosphorylation. The JAK-STAT pathway regulates immune signaling, involving mediators such as type I interferons, interferon-γ (IFN-γ), and interleukins (IL) 2, 4, 6, 7, 9, 12, 15, 21, 23, and 27, which contribute to the pathogenesis of IBD (Sandborn et al., 2017). In 2018, Tofacitinib received approval from the U.S. Food and Drug Administration (FDA) for treating adults with moderately to severely active UC. It was later approved by the European Medicines Agency (EMA) for moderate-to-severe UC (Kucharzik et al., 2020). The American Gastroenterological Association (AGA) also recommends Tofacitinib for inducing and maintaining remission in UC patients (American Gastroenterological Association, 2020). Given its significant clinical benefits and favorable prognosis, Tofacitinib has become a focal point of current research. Conducting a rounded analysis of Tofacitinib-related studies can supply valuable insights into its therapeutic advancements for UC.

CiteSpace is a knowledge mapping tool built on co-citation analysis and path network algorithms. It visualizes the structure of knowledge in specific fields and tracks the evolution of knowledge communities through data mining, information analysis, and mapping (Chen, 2004). Through the past few decades, many researchers and organizations have conducted clinical studies on Tofacitinib for UC. This study utilizes the core literature from the Web of Science (WOS) database and employs CiteSpace to visualize and construct a knowledge map, revealing the current state, research hotspots, and developmental trends of Tofacitinib research in UC. These findings aim to provide a foundation for future research directions.

## 2 Methods

### 2.1 Data sources and search strategy

**FIGURE 1:**
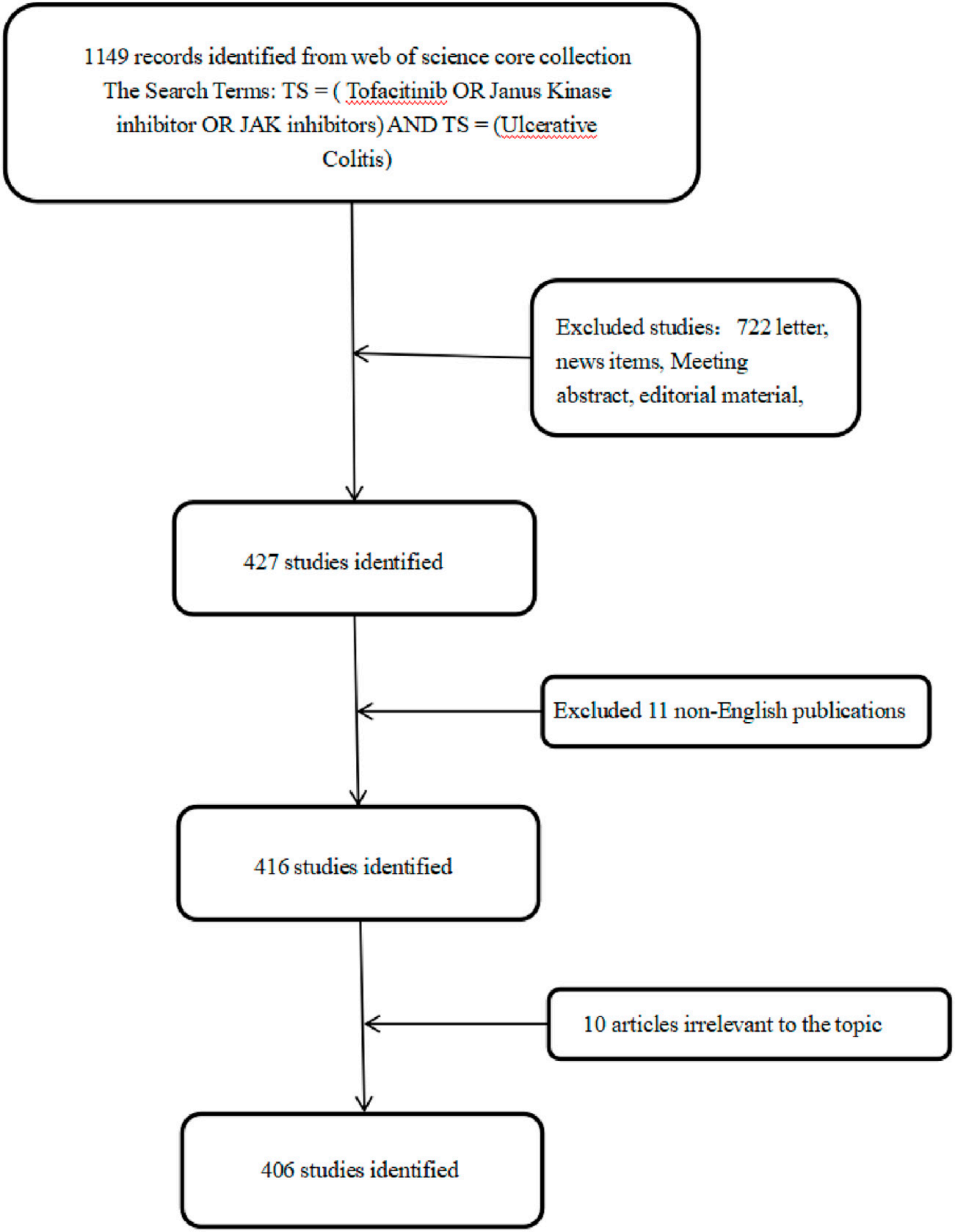
The flowchart of the literature screening process in this study.

WOS is a web-based academic resource platform developed by Clarivate Analytics (United States). Known for its comprehensive coverage, authoritative data, powerful search capabilities, frequent updates, and convenient access, it is widely utilized in the medical community. The core collection of WOS includes 10 sub-databases, such as the Science Citation Index (SCI), Social Sciences Citation Index (SSCI), and Arts & Humanities Citation Index (A&HCI). Among these, the Web of Science Core Collection (WoSCC) was selected for this study to search literature on Tofacitinib in the treatment of UC. Data were extracted from WoSCC on 14 November 2024. The search formula was defined as TS = (Tofacitinib OR Janus Kinase inhibitor OR JAK inhibitors) AND TS = (Ulcerative Colitis), covering publications from 1 January 2008 to 14 November 2024. A total of 1,149 articles were identified, with the flowchart of the included studies presented in Figure 1. Only articles in English were considered. To maintain objectivity and accuracy, the selection was restricted to “articles,” “early access publications,” and “book chapters.”

### 2.2 Literature inclusion criteria

#### 2.2.1 Inclusion criteria

① Studies related to Tofacitinib in the treatment of UC.
② Studies with complete metadata and accessible full texts, including information on country, organization, journal, author, and other relevant details.

#### 2.2.2 Exclusion criteria

① Studies unrelated to the research topic.
②Conference abstracts, letters, journal reviews, books, retractions, and errata.
③ Duplicate publications or studies identified as duplicates and only the most recent publication with the most comprehensive information was included.

The initial literature search and screening were conducted by two researchers. In cases of disagreement, a third researcher made the final decision. Based on the inclusion and exclusion criteria, a total of 1,149 articles were identified, screened, and evaluated. Ultimately, 406 relevant publications were exported as “full records and references” for analysis and visualization in CiteSpace.

### 2.3 Analysis tool

All valid data exported from the WoSCC database were imported into CiteSpace (v.6.3.R1) in Refworks format, followed by parameter adjustments for visualization and summary. CiteSpace is a scientific literature analysis tool jointly developed by Dr. Chaomei Chen from the College of Information Science and Technology at Drexel University in the United States and the WISE Laboratory at Dalian University of Technology. It is primarily used to analyze the latent knowledge embedded in scientific literature and to visualize the structure, patterns, and distribution of scientific knowledge (Chen et al., 2014).

## 3 Result

### 3.1 Time distribution map of literatures

**FIGURE 2:**
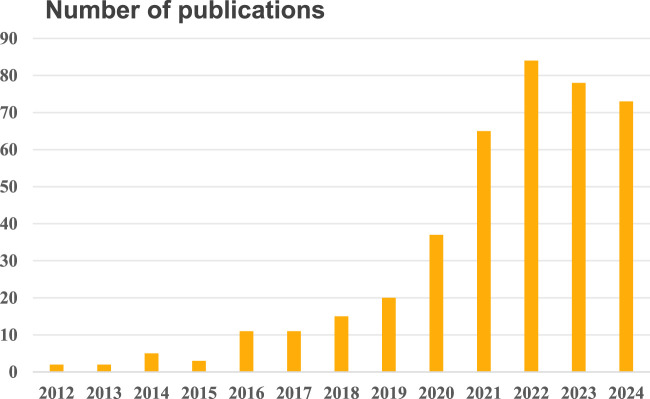
Annual publication trend on tofacitinib treatment in UC research (2012–2024).

Over the past 12 years (2012–2024), there has been a significant increase in annual publications, with 406 scientific papers published in SCI journals worldwide. As illustrated in Figure 2, the number of annual publications on Tofacitinib treatment in the field of UC research shows a general upward trajectory, rising from 2 publications in 2012 to 73 in 2024, with a peak of 84 publications in 2022. The cumulative publications indicate that Tofacitinib treatment has been a major research focus over the past decade, suggesting its potential to remain a key area of interest for the scientific community in the future. Nevertheless, in spite of the overall growth in publications, the rate and extent of this increase vary annually. This variability may be influenced by factors such as research trends, environmental changes, and funding availability in this field.

### 3.2 Countries/regions and institutional distribution analysis

The time slices for the publications we analyzed were set to 1 year, with each country or institution represented by a node. The color of the nodes indicates different years, and the size of the nodes reflects their activity level, proportional to their contribution to Tofacitinib research. The lines connecting the nodes represent affiliations or collaborations.

**FIGURE 3:**
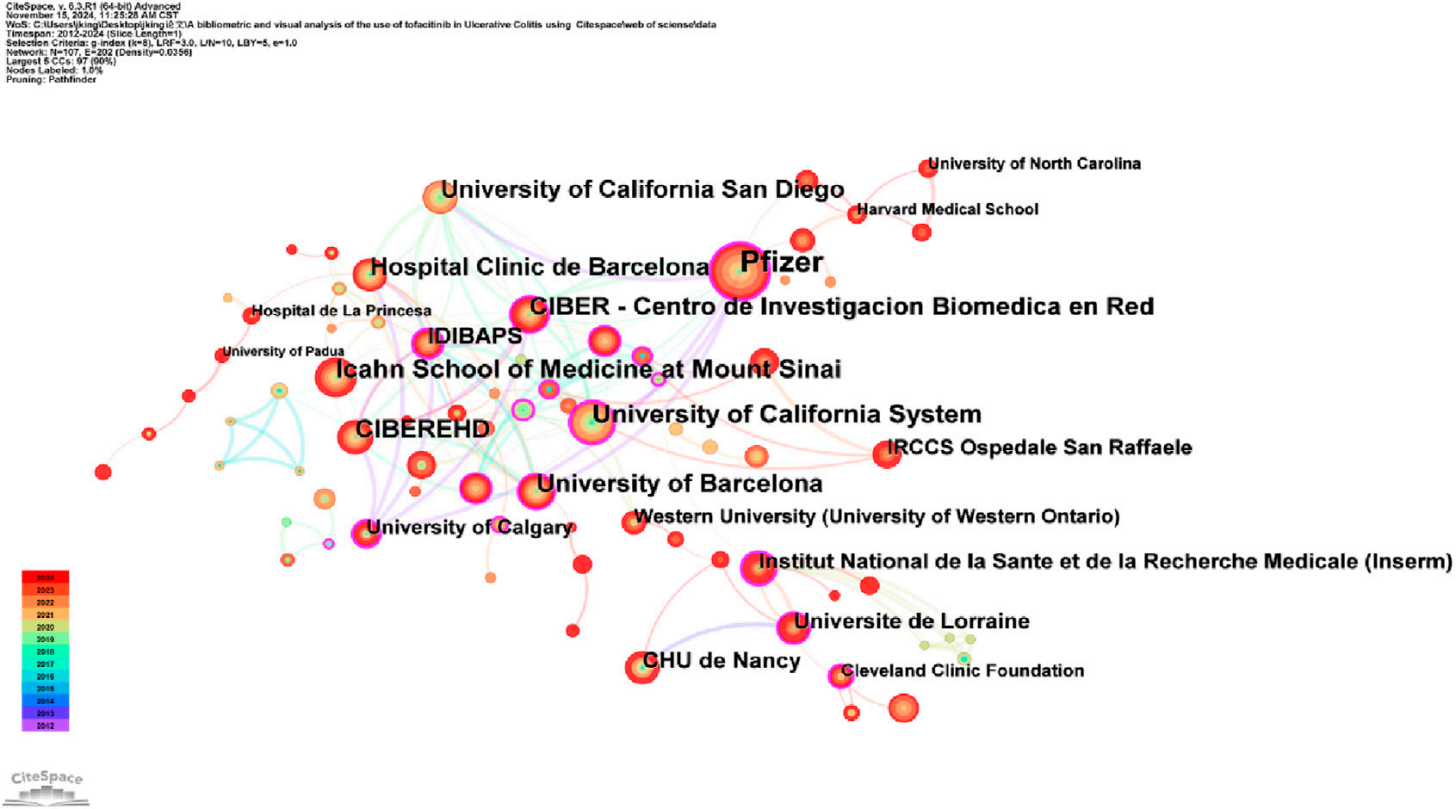
Diagram illustrating a selection of collaborative relationships between institutions from 2012 to 2024.

**FIGURE 4:**
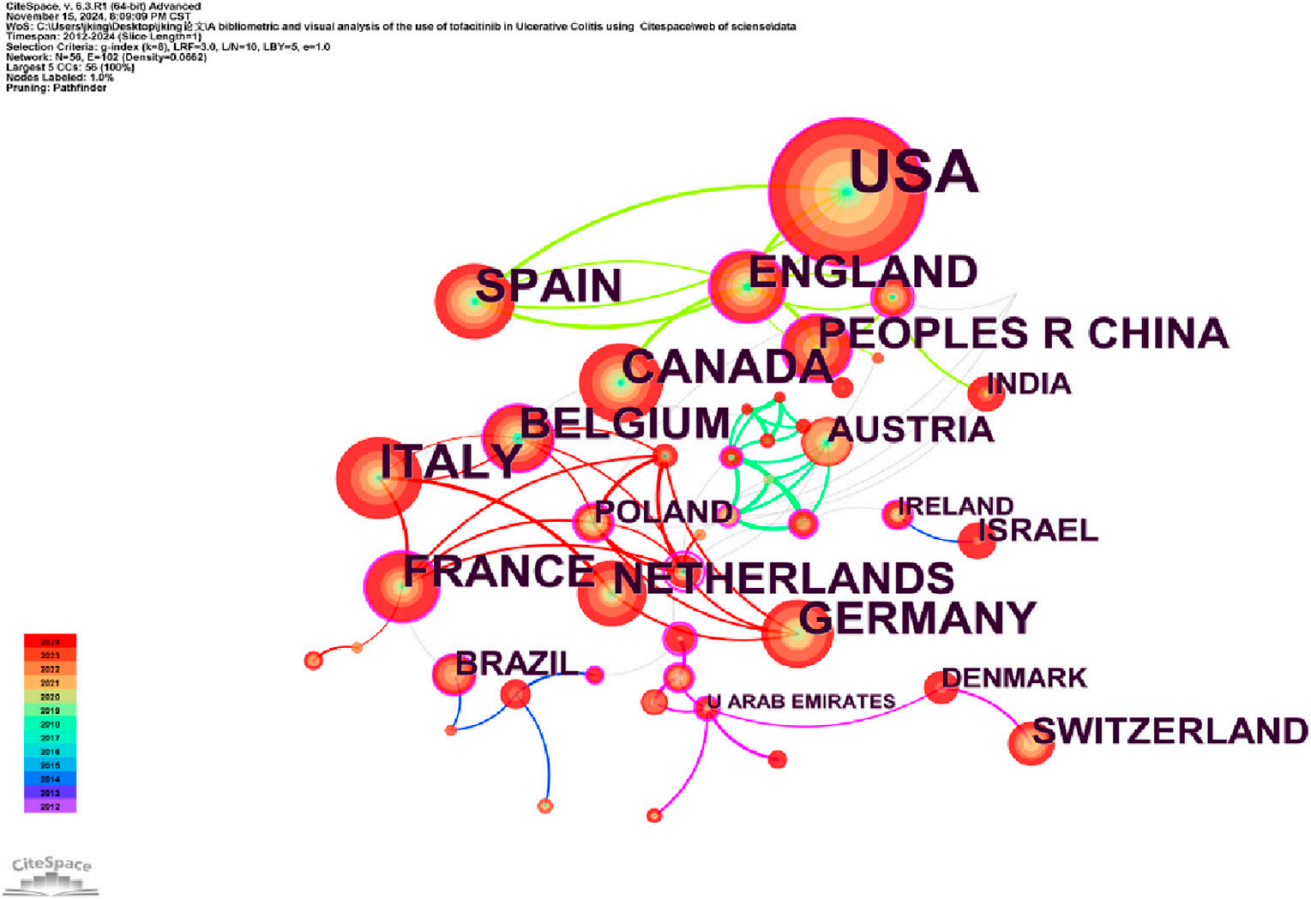
Chart depicting a portion of the cooperative relationships between countries from 2012 to 2024.

Analyzing the publishing institutions and their collaborations is crucial for identifying core institutions in this research field. Figure 3 illustrates co-institutional collaborations in Tofacitinib research. The knowledge map includes 107 institutions and 202 links, highlighting strong institutional collaborations and significant contributions from various countries. Table 1 shows that Pfizer was the most productive institution (n = 85), followed by the Icahn School of Medicine at Mount Sinai (n = 46), the University of California System (n = 44), and CIBEREHD (n = 39). Additionally, Pfizer, the University of California System, and IDIBAPS exhibit higher centrality, reflecting extensive collaboration with academic institutions. Geographically, the dominant institutions are mainly based in the United States and Spain. The widespread occurrence of UC in North America and Europe has prompted significant efforts by researchers to explore and develop novel therapeutic approaches, such as tofacitinib. Furthermore, these regions benefit from progressive study infrastructures and are home to many skilled gastroenterologists, including Su Chinyu, Danese Silvio, Sandborn William J, among others. Collaborative initiatives across various countries and institutions have led to the execution of numerous multicenter randomized controlled trials, which have compared tofacitinib to other medications, such as upadacitinib and filgotinib. These studies have contributed to a more comprehensive evaluation of tofacitinib’s clinical effectiveness, safety profile, and its real-world performance. Figure 4 shows a collaborative network of countries/regions with 56 nodes, 102 connections, and a density of 0.0662, indicating the involvement of 56 countries/regions in studying Tofacitinib’s clinical application in UC. As shown in Figure 4; Table 1, the United States (n = 199) ranked first in SCI research articles, followed by Italy (n = 63), Canada (n = 57), Spain (n = 56), and Germany (n = 46). North American and European nations lead this research field, maintaining strong collaborative ties. In contrast, publication output from Asia is comparatively lower, with Japan being the primary contributor.

**TABLE 1:** The leading 10 countries/regions of productivity, along with their respective institutions.

Rank	Country	Count	Centrality	Year	Institution	Count	Centrality	Year
1	United States	199	0.14	2012	Pfizer	85	0.36	2012
2	ITALY	63	0.03	2015	Icahn School of Medicine at Mount Sinai	46	0.07	2014
3	CANADA	57	0.06	2012	University of California System	44	0.24	2012
4	SPAIN	56	0	2012	CIBEREHD	39	0.05	2012
5	GERMANY	46	0.04	2016	CIBER - Centro de Investigacion Biomedica en Red	39	0.11	2012
6	FRANCE	45	0.3	2013	University of Barcelona	38	0.1	2012
7	ENGLAND	45	0.28	2012	University of California San Diego	36	0.09	2012
8	BELGIUM	42	0.11	2012	Hospital Clinic de Barcelona	35	0.05	2012
9	NETHERLANDS	41	0	2016	IDIBAPS	29	0.21	2012
10	JAPAN	40	0.14	2020	Mayo Clinic	27	0.03	2017

### 3.3 Authors and co-cited authors visual analysis

**FIGURE 5:**
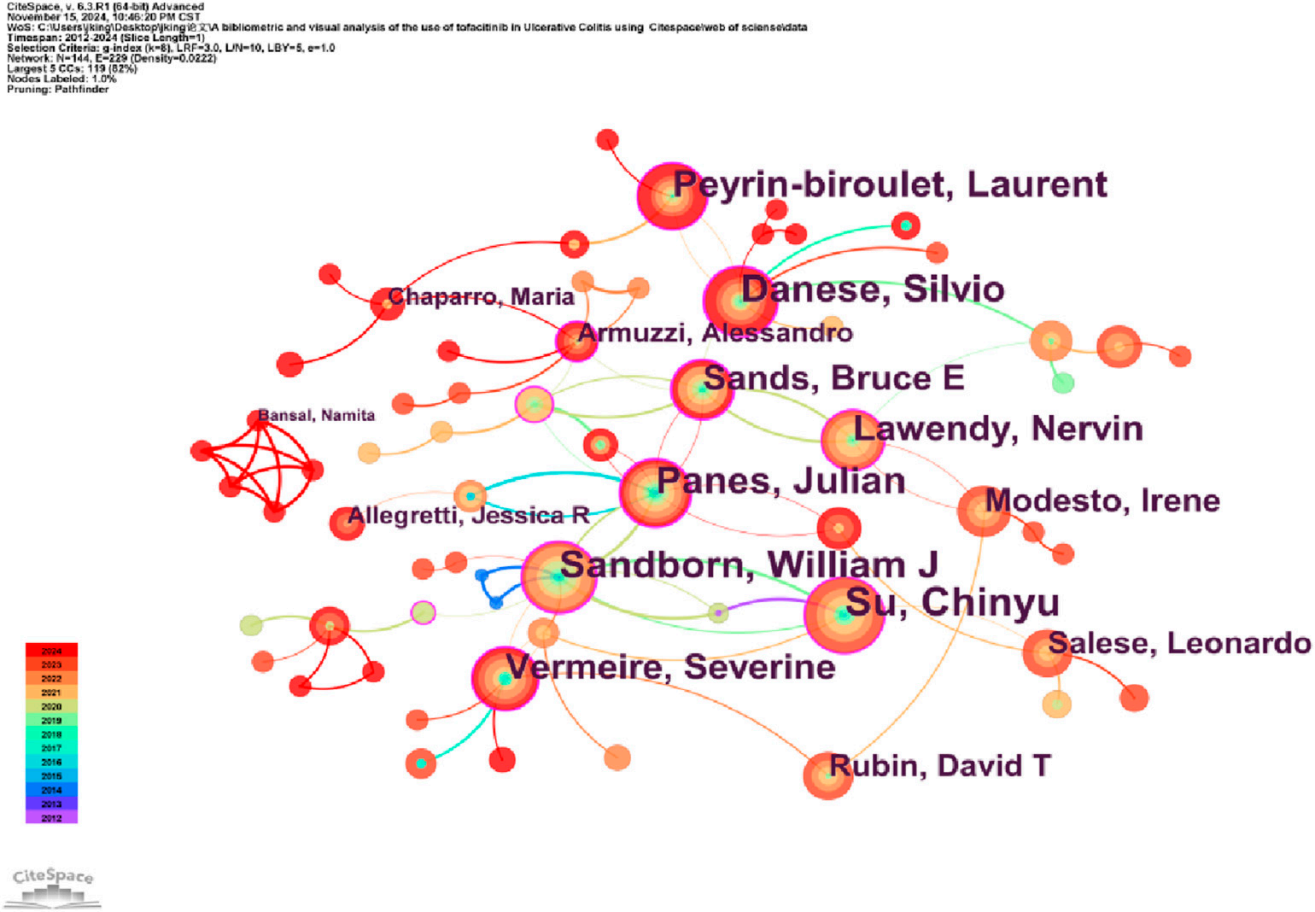
The network of author, 2012–2024.

CiteSpace generated a network graph illustrating author collaborations, showing that 144 authors published articles on Tofacitinib-related clinical research in UC patients (Figure 5). As illustrated in Table 2, Su Chinyu (Pfizer, Collegeville, PA, United States) ranks as the most productive author, with a total of 25 publications. Su Chinyu stands out as the highest producer in terms of publications, with a total of 41 papers and an H-index of 29. Her research primarily centers on the clinical applications of Tofacitinib in treating UC. Despite this, she is not ranked among the top 10 most co-cited authors in the field. In contrast, William J. Sandborn ranks third among the top 10 most productive authors, while being the most frequently co-cited. With 32 publications and an H-index of 166, he is regarded as a leading authority on UC treatment globally. Sandborn has acknowledged the promise of small molecule therapies in UC and is deeply involved in researching biologics, including ustekinumab and adalimumab, as alternative treatment options. His work includes a series of multicenter, randomized controlled trials to rigorously assess the clinical effectiveness and safety profiles of these therapeutic agents. Figure 5, generated by CiteSpace, presents author collaborations in Tofacitinib-related studies. Su Chinyu, William J. Sandborn, Julian Panés, et al. collaborated extensively, with their partnerships playing a crucial role in advancing comprehensive research in the field. Their studies aim to investigate the underlying causes of UC and assess the clinical effectiveness and safety of Tofacitinib in real-world settings across national clinical environments.

**TABLE 2:** The top 10 most productive authors and co-cited authors.

Rank	Author	Publications	Citations	H-index	Country	Rank	Co-cited author	Co-citations	Centrality	H-index
1	Su, Chinyu	41	5,133	29	United States	1	Sandborn, William J	314	0.00	166
2	Danese, Silvio	32	7,723	38	Italy	2	Sands, Bruce E	142	0.01	92
3	Sandborn, William J	32	116,188	166	United States	3	Feagan, Brian G	127	0.09	113
4	Panes, Julian	31	34,388	93	Spain	4	Danese, Silvio	118	0.02	38
5	Peyrin-biroulet, Laurent	29	36,868	86	France	5	Rubin, David T	79	0.02	67
6	Vermeire, Severine	25	79,507	135	Belgium	6	Vermeire, Severine	75	0.01	135
7	Sands, Bruce E	24	46,570	92	United States	7	Panes, Julian	75	0.03	93
8	Lawendy, Nervin	24	2,879	18	United States	8	Singh, Siddharth	74	0.02	57
9	Modesto, Irene	17	921	18	United States	9	Rutgeerts, Paul	74	0.01	145
10	Salese, Leonardo	17	489	12	United States	10	Winthrop,KL	69	0.09	73

### 3.4 Co-cited journals and references analysis

A visual analysis indicates that a total of 151 journals have published 406 articles on tofacitinib as a treatment for UC. Table 3 provides a summary of the top 10 journals and co-cited journals, organized by publication count, co-citation frequency, corresponding IF (JCR 2023), and JCR quartile rankings. Of these journals, 50% are ranked in Q1, while 90% of the co-cited journals also belong to the same quartile. *Inflammatory Bowel Disease* has the highest number of publications (57 articles; IF-2023, 4.5; Rank: Q1), whereas *The New England Journal of Medicine* is the most frequently cited journal (344 citations; IF-2023, 96.2; Rank: Q1). A significant portion of the articles on Tofacitinib treatment for UC are cited by leading international journals, reflecting the impact of these publications. Higher citation frequencies signify a greater influence within the research domain. *Inflammatory Bowel Disease* stands out with the largest number of publications and the fourth-highest citation count, emphasizing its pivotal role in clinical research regarding Tofacitinib in UC.

**TABLE 3:** The top journal and co-cited journals.

Rank	Journal	Output	IF	JCR	Co-cited journal	Co-citations	IF	JCR
1	INFLAMM BOWEL DIS	57	4.5	Q1	NEW ENGL J MED	344	96.2	Q1
2	J CROHNS COLITIS	42	8.3	Q2	J CROHNS COLITIS	308	8.3	Q2
3	ALIMENT PHARM THER	26	7.6	Q1	GASTROENTEROLOGY	304	25.7	Q1
4	DIGEST DIS SCI	16	2.5	Q4	INFLAMM BOWEL DIS	296	4.5	Q1
5	CLIN GASTROENTEROL H	13	11.6	Q1	CLIN GASTROENTEROL H	282	11.6	Q1
6	AM J GASTROENTEROL	11	8	Q1	GUT	246	23	Q1
7	DIGEST LIVER DIS	9	4	Q3	LANCET	246	98.4	Q1
8	THER ADV GASTROENTER	9	3.9	Q2	ALIMENT PHARM THER	242	7.6	Q1
9	BMC GASTROENTEROL	8	2.5	Q3	AM J GASTROENTEROL	217	8	Q1
10	GASTROENTEROLOGY	7	25.7	Q1	ANN RHEUM DIS	122	20.3	Q1

Co-citation analysis evaluates scientific progress and identifies research frontiers. Table 4 lists the top 10 co-cited articles related to this study. The paper “Tofacitinib as Induction and Maintenance Therapy for Ulcerative Colitis,” published in the New England Journal of Medicine in 2017 as part of the Tofacitinib series, ranked first with 1,163 citations. The top 10 most co-cited publications mainly concentrate on evaluating the efficacy and safety of Tofacitinib in UC. These studies explore various aspects, including induction and maintenance therapy, real-world effectiveness of Tofacitinib for Moderate to Severe UC from numerous multicenter.

**TABLE 4:** Top 10 co-cited references related to Tofacitinib.

Title	Journal	First-author	Publication year	Total citations
Tofacitinib as Induction and Maintenance Therapy for Ulcerative Colitis	NEW ENGL J MED	Sandborn, William J	2017	1,163
Ustekinumab as Induction and Maintenance Therapy for Ulcerative Colitis	NEW ENGL J MED	Sands, Bruce E	2019	734
Safety of Tofacitinib for Treatment of Ulcerative Colitis, Based on 4.4 Years of Data From Global Clinical Trials	CLIN GASTROENTEROL H	Sandborn, William J	2019	177
ACG Clinical Guideline: Ulcerative Colitis in Adults	AM J GASTROENTEROL	Rubin, David T	2019	921
Real-world Effectiveness of Tofacitinib for Moderate to Severe Ulcerative Colitis: A Multicentre UK Experience	J CROHNS COLITIS	Honap, Sailish	2020	86
Ulcerative colitis	Lancet	Ungaro, Ryan	2017	2,019
STRIDE-II: An Update on the Selecting Therapeutic Targets in Inflammatory Bowel Disease (STRIDE) Initiative of the International Organization for the Study of IBD (IOIBD): Determining Therapeutic Goals for Treat-to-Target strategies in IBD	GASTROENTEROLOGY	Dan Turner	2021	1,248
Vedolizumab versus Adalimumab for Moderate-to-Severe Ulcerative Colitis	NEW ENGL J MED	Sands, Bruce E	2019	462
Upadacitinib as induction and maintenance therapy for moderately to severely active ulcerative colitis: results from three phase 3, multicentre, double-blind, randomised trials	Lancet	Danese, Silvio	2022	253
Tofacitinib in Ulcerative Colitis: Real-world Evidence From the ENEIDA Registry	J CROHNS COLITIS	Chaparro, Maria	2021	71

### 3.5 The analysis of keywords and burst detection

#### 3.5.1 Keywords analysis

**FIGURE 6:**
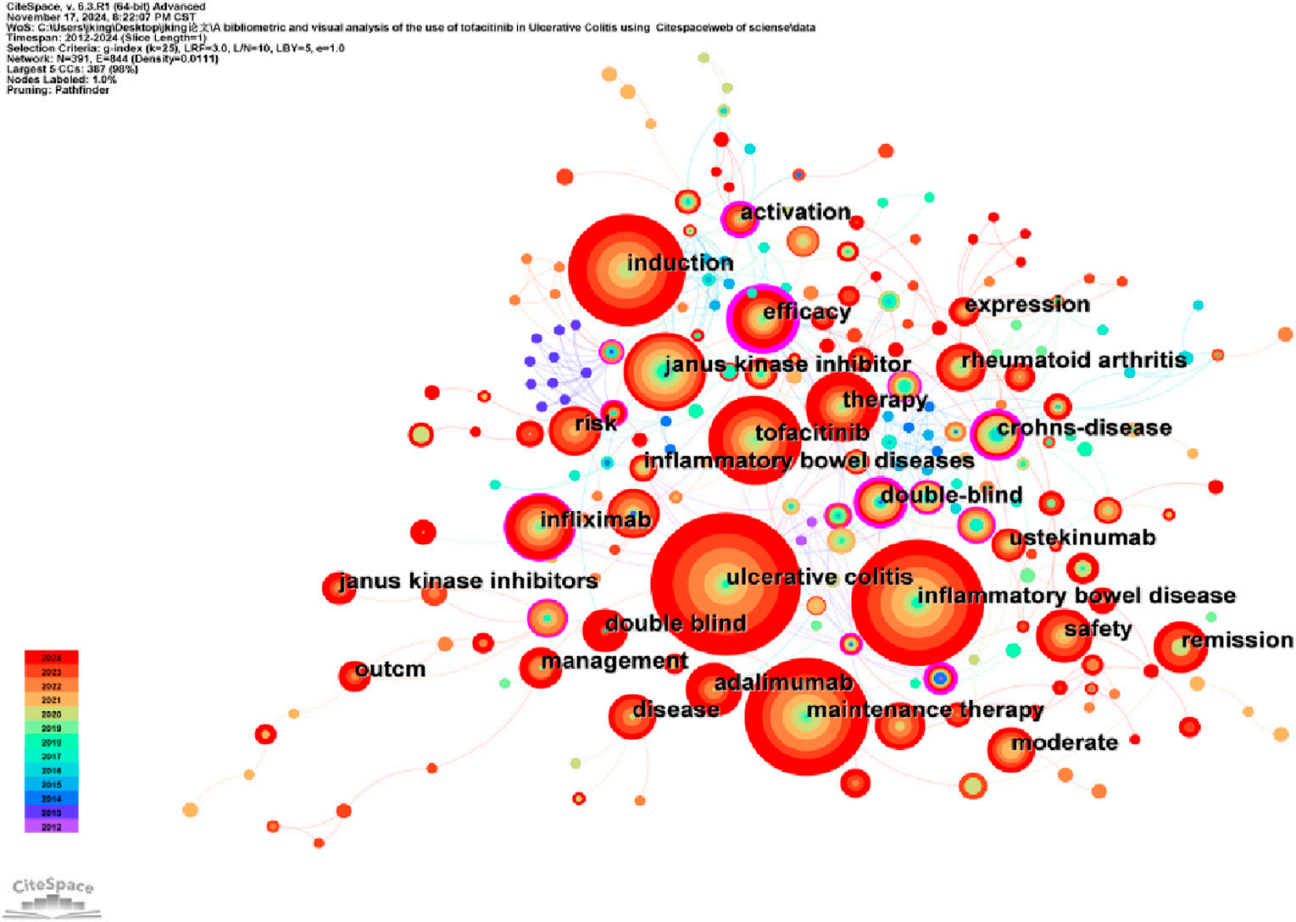
The keyword visualization map.

**FIGURE 7:**
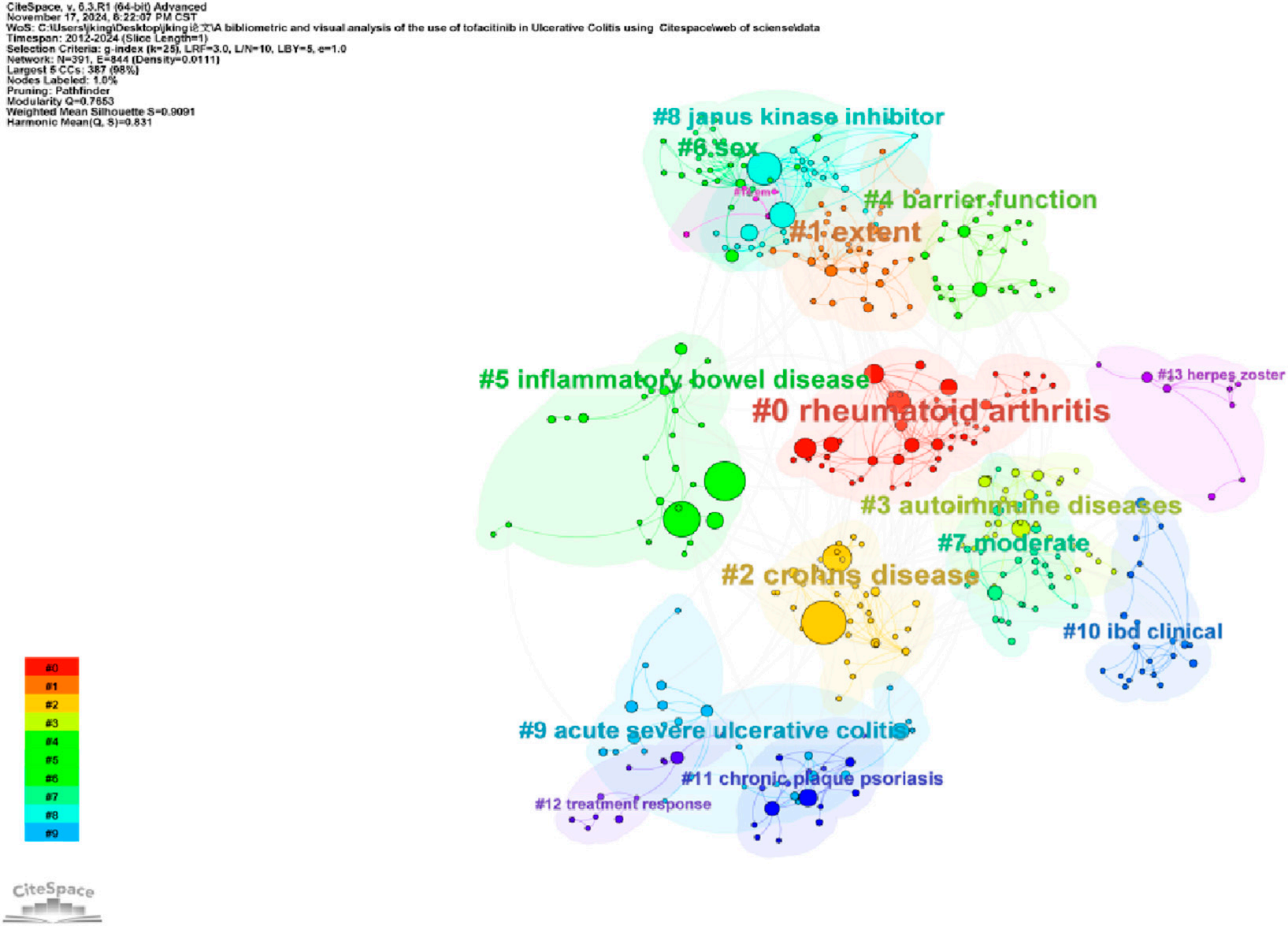
The cluster view map of keyword.

**FIGURE 8:**
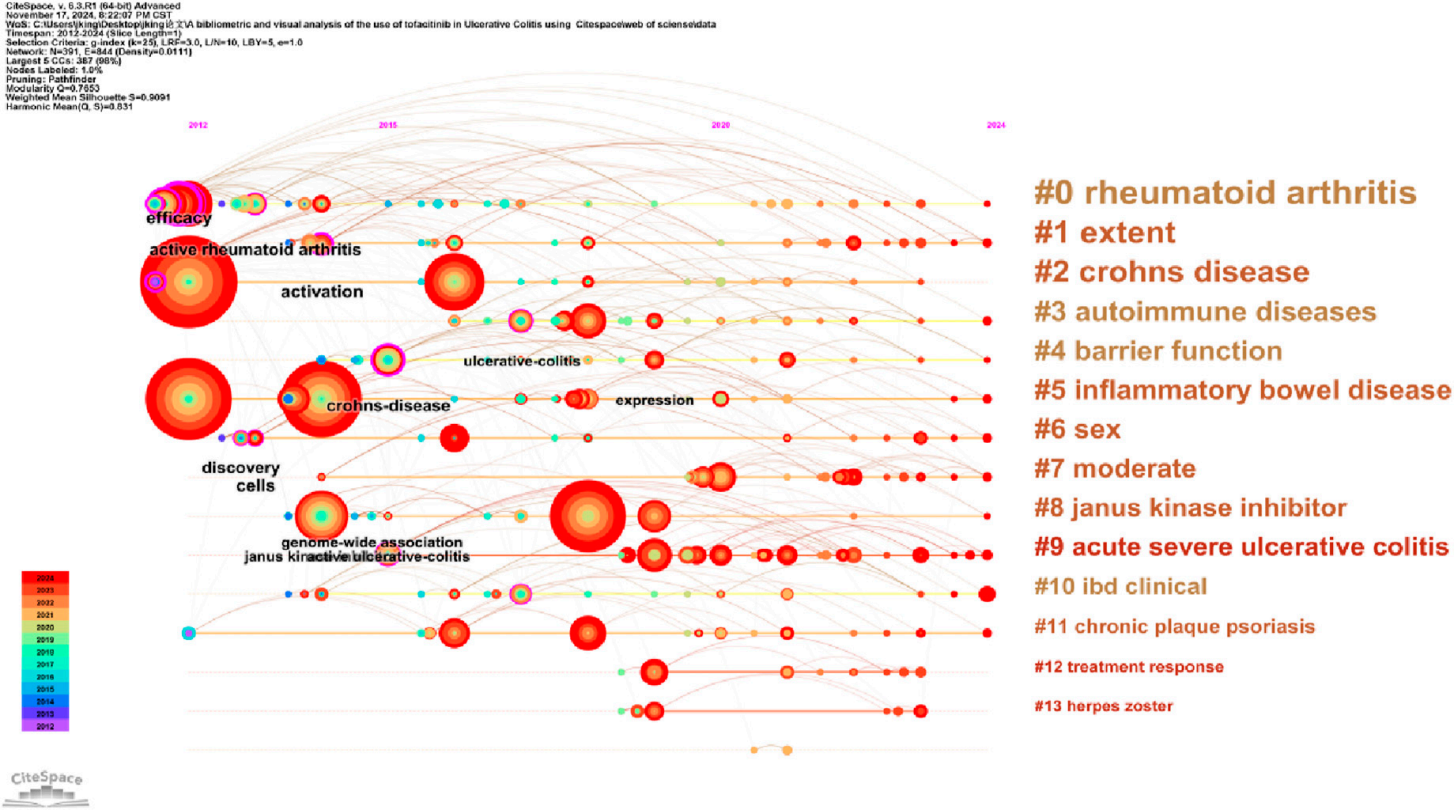
The cluster timeline view map of keywords analysis.

Keywords encapsulate the main subject matter of an article, representing its academic themes, topics, and areas of research focus. Analyzing keywords helps to identify key research trends and emerging directions within the field. CiteSpace visualizes keyword co-occurrence networks in two distinct formats: Cluster View and Timeline View. Combining keyword co-occurrence mapping and the high-frequency keyword table (Table 5; Figure 6), frequently occurring keywords include “ulcerative colitis” (219), “inflammatory bowel disease” (184), “maintenance therapy” (152), “induction” (132), “Tofacitinib” (92). The keyword clustering map groups similar keywords to identify specific research areas and topics. The timeline view introduces a time dimension in the network analysis, with color or line intensity reflecting the temporal development, thereby understanding the historical evolution of a keyword or theme. Figure 7 shows keywords clustered into 14 categories, numbered 0 to 13, with significant overlap among clusters. Figure 8 displays the timeline view, providing a visual overview of the shifting research hotspots and the developmental path of tofacitinib in UC research. Initially, tofacitinib was used for rheumatoid arthritis treatment, and since 2011, it has been explored as a therapeutic option for UC. The key terms “treatment response” and “herpes zoster” reflect the current areas of focus in the field. UC patients undergoing tofacitinib therapy may experience adverse reactions, such as herpes zoster, following standard induction or maintenance dosing. When combined with the clustering map and timeline, #3, #5, #6, #7, #8, and #10 continue to represent significant research hotspots. Recent studies have concentrated mainly on assessing the clinical efficacy and safety of tofacitinib in patients with moderate-to-severe UC, the effectiveness of combination and comparative therapies, and its role in maintaining the intestinal barrier.

**TABLE 5:** The top 10 keywords.

Rank	Keywords	Count	Centrality	Year
1	Ulcerative colitis	219	0	2012
2	Inflammatory bowel disease	184	0	2012
3	Maintenance therapy	152	0.02	2014
4	Induction	132	0	2018
5	Tofacitinib	92	0	2016
6	Janus kinase inhibitor	78	0.09	2014
7	Crohns disease	66	0.46	2012
8	Therapy	58	0.08	2012
9	Infliximab	50	0.13	2012
10	Efficacy	44	0.43	2012

#### 3.5.2 Burst detection

**FIGURE 9:**
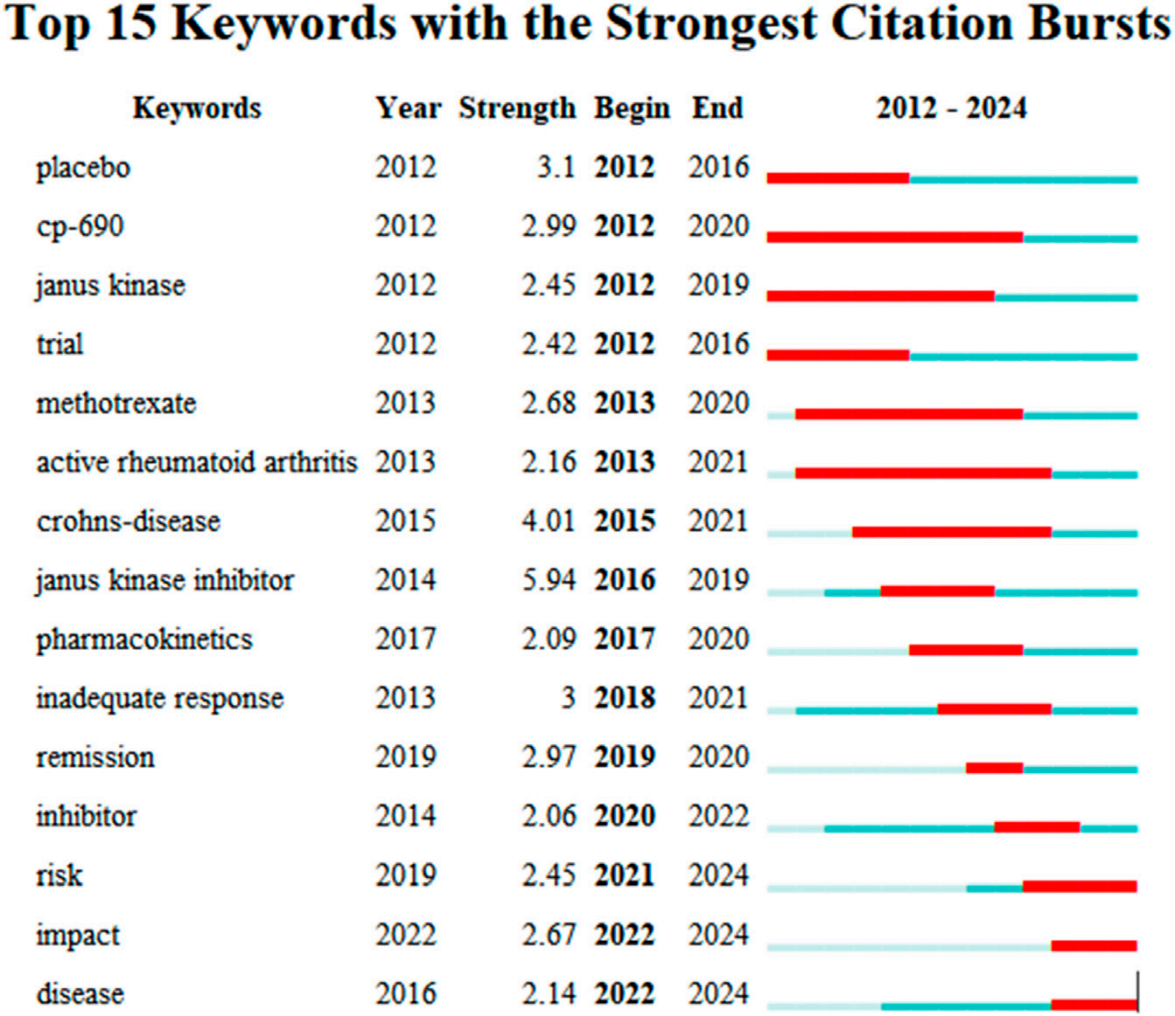
The top 15 keywords with the most significant citation burst.

Kleinberg’s burst detection algorithm is a core function of CiteSpace, used to identify turning points in the popularity of keywords or references within specific time periods. In Figure 9, The blue line indicates the time period, while the red line shows the burst duration. Keywords with the strongest burst intensities include “Janus kinase inhibitor” (5.94), “Crohn’s disease” (4.01), and “placebo” (3.1), demonstrating their prominent relevance to researchers during the specific time intervals.

## 4 Discussion

### 4.1 General information

This study employed the WoS database to proceed a full-scale literature search on Tofacitinib treatment for UC, focusing on publications from 2012 to 2024.The analysis included 406 Tofacitinib-related articles from 151 institutions, published between 1 January 2012, and 14 November 2024, in the WOSCC database. With contributions from 144 authors, the growing number of published articles indicates increasing attention to Tofacitinib. In 2012, the first article on Tofacitinib treatment, titled “Tofacitinib, an oral Janus kinase inhibitor, in active ulcerative colitis” by William J. Sandborn et al., was published, initiating research on its use in UC treatment. In the past decades, studies on Tofacitinib treatment has expanded significantly.

Table 1 shows that the United States and Italy have made significant contributions to Tofacitinib treatment UC. The United States leads in the number of published articles, with Italy and Canada following closely behind. These three countries are among the top 10 in terms of publication volume (Table 1). Of the top 10 research organizations, five are from the United States and five from Spain, with Pfizer (0.36) having the highest centrality. Additionally, 56 countries and 107 institutions worldwide are engaged in Tofacitinib research for UC, but limited collaboration and information exchange between groups may hinder progress. Collaboration with international institutions should be encouraged to advance Tofacitinib research in UC, generating real-world data from diverse populations. Regarding authorship and co-citation, Su Chinyu from Pfizer United States (41 articles) was the most prolific author. William J. Sandborn from the University of California, San Diego, has the highest co-citation frequency and an H-index of 166. The top three most-published authors, Su Chinyu, Silvio Danese, and William J. Sandborn, co-authored 13 articles on Tofacitinib in UC treatment, including a study demonstrating Tofacitinib’s consistent safety profile over 7 years (Sandborn et al., 2022).

Publishing in academic journals is a key outcome of scientific research, and analyzing journal distribution helps identify core journals in the field. According to Table 4, *Inflammatory Bowel Diseases* was the most published journal (57 articles), while *the NEW ENGL J MED* (344 co-citations) was the most cited. Cited journals were predominantly high-impact, indicating the global significance of Tofacitinib-related research.

### 4.2 Hotspots and frontiers

Keyword co-occurrence analysis, clustering, and the identification of emergent keywords have played a crucial role in elucidating the primary research themes, focal areas, and emerging hotspots in the context of tofacitinib treatment for UC. Beyond the core terms “tofacitinib” and “ulcerative colitis,” Table 5 highlights additional key concepts, such as “maintenance therapy,” “induction,” “infliximab,” and “efficacy,” which reflect the current trends and evolving themes in this area of research. Temporal analysis, visualized through a timeline view, illustrates a dynamic shift in the research landscape. Over time, there has been an increasing emphasis on the broader clinical implications of tofacitinib, with growing attention to its indications, safety profile, potential for combination therapies, comparative effectiveness with other treatments, and its influence on gut barrier function. This evolving focus underscores the expanding scope of clinical investigations into tofacitinib, as researchers continue to explore its full therapeutic potential and broader impact on UC management.

#### 4.2.1 Tofacitinib treats UC by improving intestinal barrier function

The intestinal epithelial barrier, composed of epithelial cells, acts as a physical shield preventing excessive contact between pathogenic antigens and immune cells in the lamina propria (Foerster et al., 2022). Additionally, the epithelium responds to intestinal injury by secreting factors that promote epithelial repair, initiate wound healing, and activate innate and adaptive immunity (Xavier and Podolsky, 2007). Disruption of the intestinal epithelial barrier allows foreign antigen uptake and repeated overactivation of the mucosal immune system, leading to chronic intestinal inflammation (Yao et al., 2021).

Genetic variants regulating or belonging to the JAK-STAT signaling pathway are related with IBD risk. JAK-STAT pathway inhibitors are now the effective treatment option for UC (Lei et al., 2021). Tofacitinib inhibits JAK1 and JAK3 activity, blocks signaling pathways such as IL-2, IL-4, IL-6, IL-7, IL-9, IL-15, and IL-21, reduces pro-inflammatory factor production, and enhances intestinal barrier integrity, thereby alleviating UC symptoms (Seal et al., 2023). Alexis Gonneaud et al. demonstrated that Tofacitinib restored claudin-3 levels and basolateral localization, increased Paneth cell numbers, and improved barrier function in a chronic intestinal inflammation model with IEC-Hdac1 and Hdac2 deficiency. mTOR (mammalian target of rapamycin) inactivation blocks Notch signaling in intestinal epithelial cells, suggesting that Paneth cell activation may indirectly result from mTOR and Notch inhibition (Gonneaud et al., 2021). Anica Sayoc-Becerra et al. revealed that Tofacitinib pretreatment effectively inhibited IFN-γ-induced reductions in transepithelial electrical resistance (TER) and elevations in 4 kDa FITC-dextran permeability (FD4). This intervention partially restored TER and claudin-2 expression while completely normalizing FD4 permeability and the localization of ZO-1 (Sayoc-Becerra et al., 2020). Marianne R. Spalinger et al. demonstrated that in Ptpn2-LysMCre mice, Tofacitinib corrected impaired barrier function caused by PTPN2 loss in macrophages and/or IECs, reduced colonic proinflammatory macrophages, and restored claudin-2, claudin-4, and occludin expression, thereby rescuing the barrier defect. However, Tofacitinib did not restore JAM-A levels but inhibited the secretion of inflammatory factors such as IL-6, IL-22, and TNF-α (Spalinger et al., 2021).

Understanding the JAK-STAT pathway’s role in regulating intestinal permeability is crucial for developing effective therapies to enhance epithelial barrier function and maintain intestinal homeostasis. Variations in Tofacitinib’s efficacy in addressing pore and leaky tight junction defects exist, and its enhancement of epithelial barrier function likely contributes to its benefits in IBD patients. However, studies in this area are limited to animal and *in vitro* experiments, with a lack of clinical data and small sample sizes. Further research is needed.

#### 4.2.2 Clinical response and remission

Most current studies on Tofacitinib for UC focus on moderate-to-severe cases, a refractory condition unresponsive to steroids or vedolizumab. Laura A. Lucaciu et al., in a meta-analysis of 9 studies involving 830 patients, reported that during induction therapy (496 patients, 6 studies), 51% achieved a clinical response and 31% achieved clinical remission. In maintenance therapy, 40% achieved a clinical response, and 20% achieved clinical remission (Lucaciu et al., 2021). In a real-world study, Hiromichi Shimizu et al. found that over half of the patients continued Tofacitinib therapy for 48 weeks, avoiding colectomy (Shimizu et al., 2021). These findings provide hope for patients with refractory UC. In another study on dose escalation and reduction of Tofacitinib in UC patients, Bruce E. Sands et al. reported that patients in remission who were reduced to 10 mg daily mostly maintained remission, though 25.4% lost remission by month 12. For induction responders who escalated their dose after failing 5 mg daily maintenance therapy, 49.1% achieved remission by month 12 (Sands et al., 2020). Tofacitinib has demonstrated promising results in moderate-to-severe UC during both induction and maintenance phases, offering hope for patients unresponsive to existing immunotherapies. Most current studies are real-world trials, primarily in clinical phases II and III. As of 21 November 2024, there are 35 FDA-approved studies on Tofacitinib for UC, with nine actively enrolling participants (Table 6).

**TABLE 6:** FDA-approved study of tofacitinib for ulcerative colitis in Recruitment Status (https://clinicaltrials.gov).

Study title	NCT number	Interventions	Sponsor	Study type
Evaluation of Oral Tofacitinib in Children Aged 2 to 17 Years Old Suffering From Moderate to Severe Ulcerative Colitis	NCT04624230	Drug: Tofacitinib	Pfizer	Interventional
Tofacitinib in Adult Patients With Moderate to Severe Ulcerative Colitis	NCT04424303	Drug: Tofacitinib	Pfizer	Observational
Observational Study To Assess The Effectiveness and Treatment Adherence Of Tofacitinib of Ulcerative Colitis In Clinical Practice In Sweden	NCT04338204	Drug: Tofacitinib	Pfizer	Observational
A Study of Vedolizumab With Tofacitinib in Adults With Ulcerative Colitis (UC)	NCT06095128	Drug: Vedolizumab Drug: Tofacitinib	Takeda	Interventional
Prediction Model for Response to Biologics and Small Molecular Agent for UC	NCT05186623	Drug: Vedolizumab, Ustekinumab, or Tofacitinib	Asan Medical Center	Observational
Effect of Tofacitinib on Coagulation and Platelet Function, and Its Role in Thromboembolic Events	NCT05313620	Drug: Tofacitinib Drug: Infliximab Adalimumab y Golimumab	Fundación de Investigación Biomédica - Hospital Universitario de La Princesa	Interventional
Impact of Anti-TNF, Vedolizumab and Tofacitinib on Aortic Stiffness, Carotid Intima-media Thickness and Cardiovascular Risk of Patients With Ulcerative Colitis	NCT04743518	Other: Aortic pulse wave velocity Other: carotid intima media thickness	Centre Hospitalier Universitaire, Amiens	Interventional
An Active Surveillance, Post-Authorization Study to Characterize the Safety of Tofacitinib in Patients With Moderately to Severely Active Ulcerative Colitis in the Real-World Setting Using Data From the United Registries for Clinical Assessment and Research (UR-CARE) in the European Union (EU)	NCT06469424	—	Grupo Espanol de Trabajo en Enfermedad de Crohn y Colitis Ulcerosa	Observational
Identification of Predictive Biomarkers for Response to Biologic Therapies and Tofacitinib in Inflammatory Bowel Disease	NCT03885713	Biological: Infliximab or adalimumab or golimumab or vedolizumab or ustekinumab or Tofacitinib	Fundación de Investigación Biomédica - Hospital Universitario de La Princesa	Interventional

#### 4.2.3 Adverse events

Tofacitinib received a “black box warning” from the FDA due to its association with coagulation events at a 10 mg twice-daily dose in rheumatoid arthritis patients with cardiovascular risk factors, as well as an increased risk of infections (Nash et al., 2021). Table 6 summarizes three studies investigating the safety of Tofacitinib in UC treatment. JAK inhibition is associated with lipid changes, adverse event infections, herpes zoster, major adverse cardiovascular events, and venous thromboembolism. A large cohort study of 1,220 patients reported that 8 (0.9%) treated with Tofacitinib 10 mg twice daily developed serious infections. Among 1,157 patients, the incidence rates of serious infections, herpes zoster (HZ), and non-HZ opportunistic infections (OIs) were 1.70 [1.24–2.27], 3.48 [2.79–4.30], and 0.15 [0.04–0.38], respectively (Winthrop et al., 2021). Patients receiving 20 mg daily exhibited higher infection rates compared to those on 10 mg daily. Similarly, a Tofacitinib UC clinical program (up to 7.8 years) reported an 8.2% incidence of HZ, mostly mild to moderate. Risk factors included older age, lower body weight, geographic region, and prior TNF inhibitor failure (Winthrop et al., 2018). Higher doses are likely to further increase the incidence of herpes zoster (HZ) (Sandborn et al., 2019). Similarly, Sands BE et al. observed an increase in non-melanoma skin cancer (NMSC) incidence in the Tofacitinib UC study, although the overall NMSC incidence remained low. Similar to herpes zoster, advanced age, prior NMSC, and TNFi failure are known risk factors for NMSC in UC patients (Sands et al., 2022). Overall, as an immunosuppressant, Tofacitinib impacts immune function while treating UC, necessitating careful monitoring of adverse events during clinical use.

### 4.3 Research gaps and recommendations for future work

Despite the increasing interest in Tofacitinib-related clinical studies, significant gaps and unresolved challenges remain in current research. First, additional large-scale, long-term follow-up studies are necessary to evaluate the long-term safety and efficacy of Tofacitinib, particularly in real-world settings. Second, the therapeutic responses and adverse effects of Tofacitinib in various patient groups (e.g., Asian patients, elderly patients, or those with co-morbidities) remain insufficiently understood. Furthermore, no uniform consensus has been reached regarding the optimal dose and treatment regimen of Tofacitinib for different disease stages (e.g., mild, moderate, or severe UC). Moreover, the potential benefits and risks of combining Tofacitinib with other therapeutic agents warrant further investigation. Studies addressing specific safety concerns, such as herpes zoster and thrombosis during treatment, remain limited.

Future research should prioritize the following areas: (1) conducting multicenter, long-term follow-up studies to comprehensively assess the safety, tolerability, and impact of Tofacitinib on the quality of life of UC patients; (2) exploring personalized therapeutic strategies by identifying biomarkers predictive of sensitivity or resistance to Tofacitinib through the integration of patients’ genomic, microbiomic, and immunological profiles; (3) investigating the molecular mechanisms of Tofacitinib in intestinal barrier repair to establish a theoretical foundation for combination therapy; (4) evaluating the potential of combining Tofacitinib with novel biologics or small molecule drugs to enhance therapeutic outcomes in refractory or recurrent UC patients. These efforts aim to optimize Tofacitinib’s application in UC treatment and improve long-term patient outcomes.

### 4.4 Limitation

Although CiteSpace is widely used for bibliometric studies, certain limitations are inevitable. While the WoSCC database is one of the most comprehensive and reliable sources for bibliometric analysis, relying solely on it and excluding other databases may introduce bias. Using CiteSpace alone for literature visualization and analysis involves a high degree of subjectivity in threshold selection. Future studies can integrate multiple visualization tools to analyze data from diverse perspectives and corroborate findings, ensuring the objectivity of results. Nevertheless, this study included most available articles on Tofacitinib and UC, and the resulting visual analysis offers scholars insights into key research hotspots, developments, and trends in the field.

## 5 Conclusion

Tofacitinib has received approval from both the FDA and EMA for the treatment of moderate-to-severe UC, particularly for adult patients who have not responded to conventional therapies, failed previous treatments, or are intolerant to them. In the global context, the United States and Europe have been at the forefront of clinical research on UC, making substantial contributions to advancing the field. At the same time, there is a call for enhanced cross-border collaboration and information exchange, particularly in Asia and Africa, where the incidence of UC is rising in developing regions. Notable contributors to this field include Su Chinyu and William J. Sandborn. The substantial academic interest in the use of Tofacitinib for UC is evidenced by the high citation rate of related articles in prominent international journals. Current research underscores that Tofacitinib is an effective and safe treatment option for refractory UC, particularly in patients with moderate-to-severe disease who are unresponsive to corticosteroids or vedolizumab. By reducing the need for colectomy, Tofacitinib shows significant potential in the clinical management of UC. However, it is important to consider the adverse effects associated with the drug and the potential harm these may cause to patients. Overall, Tofacitinib demonstrates substantial promise for future clinical applications in the treatment of UC.

## Data Availability

The original contributions presented in the study are included in the article/Supplementary material, further inquiries can be directed to the corresponding authors.

## References

[B1] American Gastroenterological Association (2020). Pharmacological management of adult Outpatients with moderate to severely active ulcerative colitis: clinical decision support tool. Gastroenterology 158, 1462–1463. 10.1053/j.gastro.2020.03.011 32216899

[B2] Buie M. J. Quan J. Windsor J. W. Coward S. Hansen T. M. King J. A. (2023). Global hospitalization trends for crohn's disease and ulcerative colitis in the 21st century: a systematic review with temporal analyses. Clin. Gastroenterol. Hepatol. 21, 2211–2221. 10.1016/j.cgh.2022.06.030 35863682

[B3] Chen C. (2004). Searching for intellectual turning points: progressive knowledge domain visualization. Proc. Natl. Acad. Sci. U. S. A. 101 (Suppl. 1), 5303–5310. 10.1073/pnas.0307513100 14724295 PMC387312

[B4] Chen C. Dubin R. Kim M. C. (2014). Emerging trends and new developments in regenerative medicine: a scientometric update (2000 - 2014). Expert Opin. Biol. Ther. 14, 1295–1317. 10.1517/14712598.2014.920813 25077605

[B5] Fan B. Wang M. (2023). Tofacitinib in recalcitrant bullous pemphigoid: a report of seven cases. Br. J. Dermatol 188, 432–434. 10.1093/bjd/ljac078 36763722

[B6] Foerster E. G. Mukherjee T. Cabral-Fernandes L. Rocha J. D. B. Girardin S. E. Philpott D. J. (2022). How autophagy controls the intestinal epithelial barrier. Autophagy 18, 86–103. 10.1080/15548627.2021.1909406 33906557 PMC8865220

[B7] Gonneaud A. Turgeon N. Boisvert F. M. Boudreau F. Asselin C. (2021). JAK-STAT pathway inhibition partially restores intestinal homeostasis in Hdac1-and hdac2-intestinal epithelial cell-deficient mice. Cells 10, 224. 10.3390/cells10020224 33498747 PMC7911100

[B8] Gros B. Kaplan G. G. (2023). Ulcerative colitis in adults: a review. Jama 330, 951–965. 10.1001/jama.2023.15389 37698559

[B9] Khanna D. Padilla C. Tsoi L. C. Nagaraja V. Khanna P. P. Tabib T. (2022). Tofacitinib blocks IFN-regulated biomarker genes in skin fibroblasts and keratinocytes in a systemic sclerosis trial. JCI Insight 7, e159566. 10.1172/jci.insight.159566 35943798 PMC9536259

[B10] Kobayashi T. Siegmund B. Le Berre C. (2020b). Ulcerative colitis. London, United Kindom: Nature Reviews Disease Primers. 6, 73. 10.1038/s41572-020-00215-4 32913180

[B11] Kobayashi T. Siegmund B. Le Berre C. Wei S. C. Ferrante M. Shen B. (2020a). Ulcerative colitis. Nat. Rev. Dis. Prim. 6, 74. 10.1038/s41572-020-0205-x 32913180

[B12] Krugliak C. N. Torres J. Rubin D. T. (2022). What does disease progression look like in ulcerative colitis, and how might it Be prevented? Gastroenterology 162, 1396–1408. 10.1053/j.gastro.2022.01.023 35101421

[B13] Kucharzik T. Koletzko S. Kannengiesser K. Dignass A. (2020). Ulcerative colitis-diagnostic and therapeutic algorithms. Dtsch. Arztebl Int. 117, 564–574. 10.3238/arztebl.2020.0564 33148393 PMC8171548

[B14] Le Berre C. Honap S. Peyrin-Biroulet L. (2023). Ulcerative colitis. Lancet 402, 571–584. 10.1016/s0140-6736(23)00966-2 37573077

[B15] Lei H. Crawford M. S. McCole D. F. (2021). JAK-STAT pathway regulation of intestinal permeability: pathogenic roles and therapeutic opportunities in inflammatory bowel disease. Pharm. (Basel). 14, 840. 10.3390/ph14090840 PMC8466350 34577540

[B16] Lucaciu L. A. Constantine-Cooke N. Plevris N. Siakavellas S. Derikx L. Jones G. R. (2021). Real-world experience with tofacitinib in ulcerative colitis: a systematic review and meta-analysis. Ther. Adv. Gastroenterol. 14, 17562848211064004. 10.1177/17562848211064004 PMC8721385 34987608

[B17] Nash P. Kerschbaumer A. Dörner T. Dougados M. Fleischmann R. M. Geissler K. (2021). Points to consider for the treatment of immune-mediated inflammatory diseases with Janus kinase inhibitors: a consensus statement. Ann. Rheum. Dis. 80, 71–87. 10.1136/annrheumdis-2020-218398 33158881 PMC7788060

[B18] Rimar D. Awisat A. Kaly L. Slobodin G. Rosner I. Rozenbaum M. (2022). Response to: 'Tofacitinib for the treatment of polyarteritis nodosa: a literature review'. Correspondence on 'Tofacitinib for polyarteritis nodosa: a tailored therapy' by Rimar et al. Ann. Rheum. Dis. 81, e205. 10.1136/annrheumdis-2020-218790 32907803

[B19] Sandborn W. J. Lawendy N. Danese S. Su C. Loftus E. V. Jr. Hart A. (2022). Safety and efficacy of tofacitinib for treatment of ulcerative colitis: final analysis of OCTAVE Open, an open-label, long-term extension study with up to 7.0 years of treatment. Aliment. Pharmacol. Ther. 55, 464–478. 10.1111/apt.16712 34854095 PMC9300081

[B20] Sandborn W. J. Panés J. D'Haens G. R. Sands B. E. Su C. Moscariello M. (2019). Safety of tofacitinib for treatment of ulcerative colitis, based on 4.4 Years of data from global clinical trials. Clin. Gastroenterol. Hepatol. 17, 1541–1550. 10.1016/j.cgh.2018.11.035 30476584

[B21] Sandborn W. J. Su C. Panes J. (2017). Tofacitinib as induction and maintenance therapy for ulcerative colitis. N. Engl. J. Med. 377, 496–497. 10.1056/NEJMc1707500 28767341

[B22] Sands B. E. Armuzzi A. Marshall J. K. Lindsay J. O. Sandborn W. J. Danese S. (2020). Efficacy and safety of tofacitinib dose de-escalation and dose escalation for patients with ulcerative colitis: results from OCTAVE Open. Aliment. Pharmacol. Ther. 51, 271–280. 10.1111/apt.15555 31660640 PMC9328429

[B23] Sands B. E. Long M. D. Reinisch W. Panés J. Loftus E. V. Nduaka C. I. (2022). Tofacitinib for the treatment of ulcerative colitis: analysis of nonmelanoma skin cancer rates from the ulcerative colitis clinical program. Inflamm. Bowel Dis. 28, 234–245. 10.1093/ibd/izab056 33742652 PMC8804509

[B24] Sayoc-Becerra A. Krishnan M. Fan S. Jimenez J. Hernandez R. Gibson K. (2020). The JAK-inhibitor tofacitinib rescues human intestinal epithelial cells and colonoids from cytokine-induced barrier dysfunction. Inflamm. Bowel Dis. 26, 407–422. 10.1093/ibd/izz266 31751457 PMC7012302

[B25] Seal R. Schwab L. S. U. Chiarolla C. M. Hundhausen N. Klose G. H. Reu-Hofer S. (2023). Delayed and limited administration of the JAKinib tofacitinib mitigates chronic DSS-induced colitis. Front. Immunol. 14, 1179311. 10.3389/fimmu.2023.1179311 37275854 PMC10235777

[B26] Shimizu H. Fujii T. Hibiya S. Motobayashi M. Suzuki K. Takenaka K. (2021). Rapid prediction of 1-year efficacy of tofacitinib for treating refractory ulcerative colitis. Intest. Res. 19, 115–118. 10.5217/ir.2020.00030 32516866 PMC7873398

[B27] Smolen J. S. Landewé R. B. M. Bergstra S. A. Kerschbaumer A. Sepriano A. Aletaha D. (2023). EULAR recommendations for the management of rheumatoid arthritis with synthetic and biological disease-modifying antirheumatic drugs: 2022 update. Ann. Rheum. Dis. 82, 3–18. 10.1136/ard-2022-223356 36357155

[B28] Spalinger M. R. Sayoc-Becerra A. Ordookhanian C. Canale V. Santos A. N. King S. J. (2021). The JAK inhibitor tofacitinib rescues intestinal barrier defects caused by disrupted epithelial-macrophage interactions. J. Crohns Colitis 15, 471–484. 10.1093/ecco-jcc/jjaa182 32909045 PMC7944512

[B29] Ungaro R. Mehandru S. Allen P. B. Peyrin-Biroulet L. Colombel J. F. (2017). Ulcerative colitis. Lancet 389, 1756–1770. 10.1016/s0140-6736(16)32126-2 27914657 PMC6487890

[B30] Voelker R. (2024). What is ulcerative colitis? Jama 331, 716. 10.1001/jama.2023.23814 38306113

[B31] Wangchuk P. Yeshi K. Loukas A. (2024). Ulcerative colitis: clinical biomarkers, therapeutic targets, and emerging treatments. Trends Pharmacol. Sci. 45, 892–903. 10.1016/j.tips.2024.08.003 39261229

[B32] Winthrop K. L. Loftus E. V. Baumgart D. C. Reinisch W. Nduaka C. I. Lawendy N. (2021). Tofacitinib for the treatment of ulcerative colitis: analysis of infection rates from the ulcerative colitis clinical programme. J. Crohns Colitis 15, 914–929. 10.1093/ecco-jcc/jjaa233 33245746 PMC8218715

[B33] Winthrop K. L. Melmed G. Y. Vermeire S. Long M. D. Chan G. Pedersen R. D. (2018). Herpes zoster infection in patients with ulcerative colitis receiving tofacitinib. Inflamm. Bowel Dis. 24, 2258–2265. 10.1093/ibd/izy131 29850873 PMC6140434

[B34] Xavier R. J. Podolsky D. K. (2007). Unravelling the pathogenesis of inflammatory bowel disease. Nature 448, 427–434. 10.1038/nature06005 17653185

[B35] Yao D. Dai W. Dong M. Dai C. Wu S. (2021). MUC2 and related bacterial factors: therapeutic targets for ulcerative colitis. EBioMedicine 74, 103751. 10.1016/j.ebiom.2021.103751 34902790 PMC8671112

